# Using SCORE2 with a risk chart or online calculator: impact on model performance, treatment eligibility, and cardiovascular disease prevention

**DOI:** 10.1093/ehjqcco/qcaf122

**Published:** 2025-10-10

**Authors:** Steven H J Hageman, Stephen Kaptoge, Mari N Gynnild, Joris Holtrop, Lisa Pennells, J William McEvoy, Martin Bobak, Andrzej Pająk, Hynek Pikhart, Abdonas Tamosiunas, Yvo M Smulders, Francois Mach, David Carballo, Ewout W Steyerberg, Jannick A N Dorresteijn, Emanuele Di Angelantonio, Angela M Wood, Frank L J Visseren

**Affiliations:** Department of Vascular Medicine, University Medical Center Utrecht, PO Box 85500, Utrecht 3508 GA, The Netherlands; Cardiovascular Epidemiology Unit, Department of Public Health and Primary Care, University of Cambridge, Cambridge CB1 8RN, UK; K.G. Jebsen Center for Cardiac Biomarkers, Institute of Clinical Medicine, University of Oslo, Norway; Department of Cardiology, St. Olav University Hospital, 7030 Trondheim, Norway; Department of Circulation and Medical Imaging, Norwegian University of Science and Technology, 7491 Trondheim, Norway; Department of Vascular Medicine, University Medical Center Utrecht, PO Box 85500, Utrecht 3508 GA, The Netherlands; Cardiovascular Epidemiology Unit, Department of Public Health and Primary Care, University of Cambridge, Cambridge CB1 8RN, UK; University of Galway and National Institute for Prevention and Cardiovascular Health, Galway H91 FF68, Ireland; Department of Epidemiology and Public Health, University College London, London WC1E 7HB, UK; RECETOX, Masaryk University, 625 00 Brno, Czech Republic; Department of Epidemiology and Population Studies, Institute of Public Health, Faculty of Health Sciences, Jagiellonian University Medical College, 31-066 Kraków, Poland; Department of Epidemiology and Public Health, University College London, London WC1E 7HB, UK; RECETOX, Masaryk University, 625 00 Brno, Czech Republic; Institute of Cardiology, Lithuanian University of Health Sciences, LT-44307 Kaunas, Lithuania; Internal Medicine, Amsterdam UMC, Amsterdam 1105 AZ, The Netherlands; Division of Cardiology, Faculty of Medicine, Geneva University Hospitals, 1205 Geneva, Switzerland; Division of Cardiology, Faculty of Medicine, Geneva University Hospitals, 1205 Geneva, Switzerland; Julius Center for Health Science and Primary Care, University Medical Center Utrecht, University of Utrecht, Utrecht 3508 GA, The Netherlands; Department of Vascular Medicine, University Medical Center Utrecht, PO Box 85500, Utrecht 3508 GA, The Netherlands; Cardiovascular Epidemiology Unit, Department of Public Health and Primary Care, University of Cambridge, Cambridge CB1 8RN, UK; Cardiovascular Epidemiology Unit, Department of Public Health and Primary Care, University of Cambridge, Cambridge CB1 8RN, UK; Department of Vascular Medicine, University Medical Center Utrecht, PO Box 85500, Utrecht 3508 GA, The Netherlands

**Keywords:** Risk prediction, Cardiovascular disease, Primary prevention, 10-year CVD risk

## Abstract

**Aims:**

Current European Cardiovascular Disease (CVD) prevention guidelines recommend 10-year risk assessment using the SCORE2 model to identify individuals eligible for preventive treatment. Risk can be estimated using conventional risk charts or online calculators, though these methods may differ in precision and treatment classification.

**Methods and results:**

Individuals without established CVD or diabetes mellitus were included from CPRD (UK, Europe’s low risk region, *n* = 977 616) and HAPIEE (Czech Republic and Poland, high risk region and Lithuania, very high risk region, *n* = 11 739). During median 8.4 years [interquartile range (IQR) 5.0–10.4], 22 898 CVD events occurred. SCORE2 risk was estimated via two methods: an online calculator (unrounded SCORE2 algorithm) and risk charts from the 2021 ESC Prevention Guidelines. Predicted risks were higher with the risk charts than with the online calculator. In the low risk region, the median 10-year risk was 4.0% (IQR 2.0–6.0) with the risk charts vs. 3.7% (IQR 2.3–5.8) with the calculator. In the high/very high-risk region, risk was 9.0% (IQR 5.0–15.0) and 8.4% (IQR 4.5–13.9), respectively. Chart-based risk assessment resulted in higher treatment eligibility (6.3% vs. 4.0% in the low risk region; 51% vs. 43% in high/very high risk region). Discrimination was higher with the online calculator: difference in C-statistic +0.010 [95% confidence interval (CI) 0.008–0.012] in low risk region and +0.008 (95% CI 0.005–0.010) in high/very high risk region. Calibration was adequate for both approaches. Assuming a 50% relative risk reduction for preventive treatment, this corresponded to 53 vs. 46 events prevented per 1000 treated in the low-risk region and 80 vs. 74 in the high/very-high-risk region (calculator vs. risk charts).

**Conclusion:**

Risk assessment using SCORE2 risk charts yields too high predicted risks and too broad treatment eligibility. By avoiding rounding of risk factors, the online calculator shows better discrimination.

## Graphical Abstract

**Graphical Abstract:**
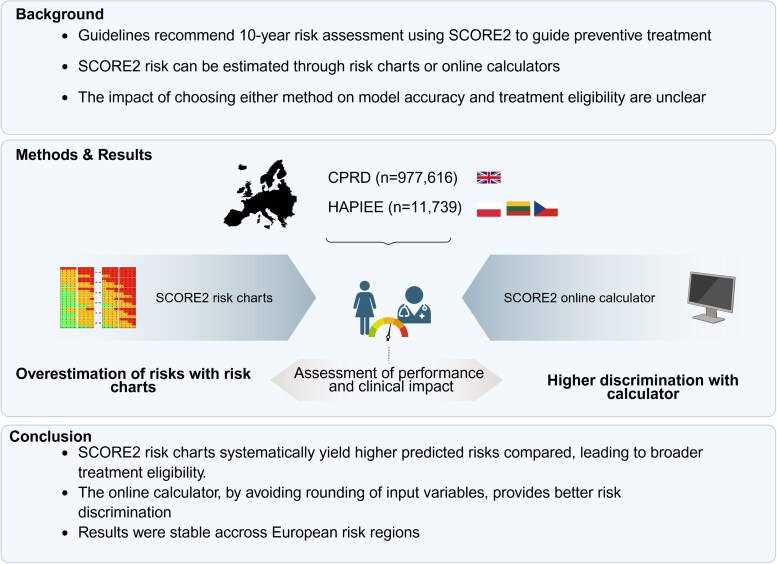

Key Learning Points What is already known:

- European Society of Cardiology (ESC) guidelines recommend SCORE2 for 10-year cardiovascular disease (CVD) risk assessment to guide preventive treatment decisions.
- SCORE2 can be applied using paper-based risk charts or an online calculator, but the potential impact of rounding and calculation precision on treatment classification is unclear.
- Accurate risk estimation is essential to ensure appropriate targeting of preventive therapies.

What this study adds:

- SCORE2 risk charts systematically yield higher predicted risks compared with the unrounded online calculator, leading to broader treatment eligibility.
- The online calculator, by avoiding rounding of input variables, provides better risk discrimination.
- Use of the calculator rather than risk charts could reduce overtreatment and maintain efficiency in preventing CVD events.

## Introduction

Cardiovascular diseases (CVD), including myocardial infarctions and strokes, are the leading cause of mortality among non-communicable diseases worldwide, accounting for approximately 18.6 million deaths in 2019.^1^ The European Society of Cardiology (ESC) CVD prevention guidelines advocate for the use of risk prediction models to improve healthcare and population-wide prevention strategies.^2^ Prediction models combine multiple CVD risk factors to estimate an individual’s 10-year risk, thereby identifying those at higher risk who may benefit most from preventive interventions.

For individuals without established CVD or diabetes mellitus, the Systematic COronary Risk Evaluation 2 (SCORE2) is the recommended model for 10-year risk assessment.^2,3^ This model can be applied using two-dimensional risk charts, provided for example in the manuscript of the 2021 ESC prevention guidelines.^2^ Additionally, risk prediction algorithms are available via online calculators, including the ESC CVD risk prediction app or the U-Prevent Medical Device. Although the risk charts are easy and convenient for use in clinical practice, they require rounding off of risk factors (for example, everybody aged between 60 and 65 years has their risk predicted with age 62.5 years as this is the midpoint for this category on the risk chart), which may affect predictive accuracy. Moreover, the risk charts use non-HDL cholesterol (HDL-c) rather than separate total and HDL-c values, potentially leading to a further imprecision in risk estimates and, possibly, systematic differences in treatment eligibility. While both the risk charts and the online calculators are used in clinical practice, their accuracy and clinical impact may differ, but this has not yet been evaluated.

Therefore, the aims of the current study were (i) to quantify the difference in model performance when applying SCORE2 as a risk chart vs. an online calculator in terms of discrimination and calibration and (ii) to quantify the clinical impact between the two methods regarding differences in treatment eligibility and expected event reduction from preventive therapy among treatment-eligible individuals.

## Methods

### Population

For the current study, individuals were included from the Clinical Practice Research Datalink (CPRD) in the UK^4^ and from the Health, Alcohol and Psychosocial factors In Eastern Europe (HAPIEE) study in Poland, Czech Republic, and Lithuania.^5^ The CPRD is a UK-based primary care database of anonymized medical records from 674 general practices, with coverage of over 11.3 million patients and is broadly representative of the general population in terms of age, sex, and ethnicity. The data used for this study were restricted to the region of England with baseline data collected between 1 April 2004 to 2006 and follow-up data to 30 November 2017. Incident nonfatal events were obtained from linkage with Hospital Episode Statistics and deaths from the Office for National Statistics. The HAPIEE study comprises prospective urban population-based cohorts from Eastern Europe, located in Krakow (Poland), Kaunas (Lithuania), and six cities of the Czech Republic. Each cohort recruited a random sample of men and women aged 45–69 years at baseline, conducted between 2002 and 2005 (2005–2008 in Lithuania), stratified by sex and 5-year age groups. From these cohorts, individuals across the SCORE2 age range of 40–69 years without prior diabetes mellitus and CVD were included.

### Statistical analysis

For all individuals, risk predictions were calculated with both implementations of the SCORE2 model, mimicking how these are applied in clinical practice. First, risk was calculated as implemented in the ESC risk prediction app or the U-Prevent Medical Device (both using the same unrounded SCORE2 algorithm), and second, using the two-dimensional risk charts published alongside the SCORE2 manuscript.^3^ For the risk charts, non-HDL-c was calculated by subtracting HDL-c from total cholesterol. Additionally, risk estimation required rounding age to the nearest 5-year group, systolic blood pressure (SBP) to 20 mmHg intervals, and non-HDL-c to 1 mmol/L intervals. Final risk values were rounded to whole number percentages. For all individuals, the SCORE2 chart of their respective region was used (CPRD as low risk region, HAPIEE Poland and Czech Republic as high risk region, HAPIEE Lithuania as very high risk region).

The primary outcome was consistent with the SCORE2 model: a composite of cardiovascular mortality, non-fatal myocardial infarction, and non-fatal stroke.^3^ Death from other causes was treated as a competing outcome in the analyses. Follow-up continued until the first non-fatal myocardial infarction, non-fatal stroke, or death or end of the event registration period.

Both approaches to applying the SCORE2 model were compared in terms of discrimination, quantified using Harrell’s C-index. Calibration was assessed by visual inspection of predicted 10-year risks vs. the observed cumulative incidence across deciles of predicted risk. Both discrimination and calibration were adjusted for competing risks to account for the possibility that individuals may die from non-cardiovascular causes before experiencing a cardiovascular event.^3,6^

Reclassification was evaluated using the net reclassification index (NRI), based on the 5% and 10% 10-year CVD risk thresholds recommended in the 2021 ESC prevention guidelines for individuals aged 50–69 years.^2^ Net reclassification index quantifies whether a model more appropriately reassigns individuals to higher or lower risk categories compared with another model. It was calculated separately for individuals who experienced an event (events) and those who did not (non-events). To enable comparison across age groups, the same treatment thresholds were applied irrespective of age. Confidence intervals (CIs) were obtained using bootstrapping (r-package *nricens*).^7,8^ Moreover, net benefit was calculated to provide an overall measure of clinical usefulness. It represents the proportion of individuals correctly identified as high-risk (true positives), adjusted for the harm of incorrectly classifying individuals as high-risk (false positives).^9,10^ This adjustment is made by applying a weighting factor that reflects the clinical trade-off between the benefit of treating a true positive and the harm of treating a false positive. In this analysis, a weighting of 1:10 was used, corresponding to a treatment threshold of 10% as recommended by clinical guidelines for individuals aged 50–69 years.^2,9^

### Treatment eligibility

Treatment eligibility was determined based on the age-specific risk thresholds for ‘Very high CVD risk’: risk factor treatment generally recommended in the 2021 ESC CVD prevention guidelines: ≥7.5% 10-year risk for individuals aged 40–49 years and ≥10% 10-year risk for individuals aged 50–69 years.^3^ Because the risk charts round to whole numbers, the effective treatment threshold in those aged 50–69 years was ≥9.5% as this is rounded upwards to 10% and marked as ‘Very high risk’ on the chart.

To estimate the impact of using the two approaches of risk estimation on CVD outcomes, a hypothetical (but in clinical practice achievable) 50% relative risk reduction was simulated for individuals considered eligible for treatment. A 50% relative risk reduction could for example be achieved by intensive lipid-lowering treatment (high-intensity statin or statin/ezetimibe combination), or with a moderate intensity statin in combination with antihypertensive treatment.^11,12^ First, the cumulative incidence of CVD events was determined in the treatment-eligible group. The cumulative incidence was then combined with a hazard ratio of 0.50 to estimate the expected event reduction.^12^

Missing data on CVD risk factors was handled using single imputation based (*aregimpute* package in R) in HAPIEE. In CPRD, with higher numbers of missing data, this was handled using multiple imputation *mice* package in R. Both methods were based on predictive mean matching, including a Nelson Aalen estimator for both CVD events and the competing outcome of non-CVD mortality. All analyses were performed with R-statistical programming (version 3.5.2, R Foundation for Statistical Computing, Vienna, Austria). The current study adheres to the TRIPOD reporting guidelines (see Supplementary material).^13^

## Results

### Study population

In total, 989 355 individuals were included with a geographic distribution covering Europe’s low CVD risk region (*n* = 977 616), the high CVD risk region (*n* = 7508), and the very high CVD risk region (*n* = 4231). Of these, 488 195 (49%) were men and the median age was 53 years [interquartile range (IQR) 46–60]. Detailed participant characteristics are presented in *Table 1*. During a median follow-up of 8.4 years (IQR 5.0–10.4), 22 898 CVD events and 22 604 non-cardiovascular deaths were observed.

**Table 1:** Baseline characteristics of the study populations

	CPRD	HAPIEE
Total participants	977 614	11 739
Risk region	Low (100%)	High (64%) Very high (36%)
Male sex	482 952 (49%)	5243 (45%)
Age (years)	53 (8)	57 (7)
Current smoker	439 022 (45%)	2990 (25%)
Systolic blood pressure (mmHg)	132 (16)	137 (20)
Total cholesterol (mmol/L)	5.5 (4.8–6.2)	5.8 (5.2–6.5)
HDL cholesterol (mmol/L)	1.4 (1.2–1.7)	1.4 (1.2–1.7)
Follow-up (years, 5th/95th percentile)	8.4 (5.0–10.4)	8.0 (6.3–12.1)
Cardiovascular events	21 772	1126
Non-CVD mortality	21 612	992

Items are shown as *N* (%), mean (SD), or median (Q1-Q3).

HDL, high-density lipoprotein; CVD, cardiovascular disease.

### Model performance

**Figure 1:**
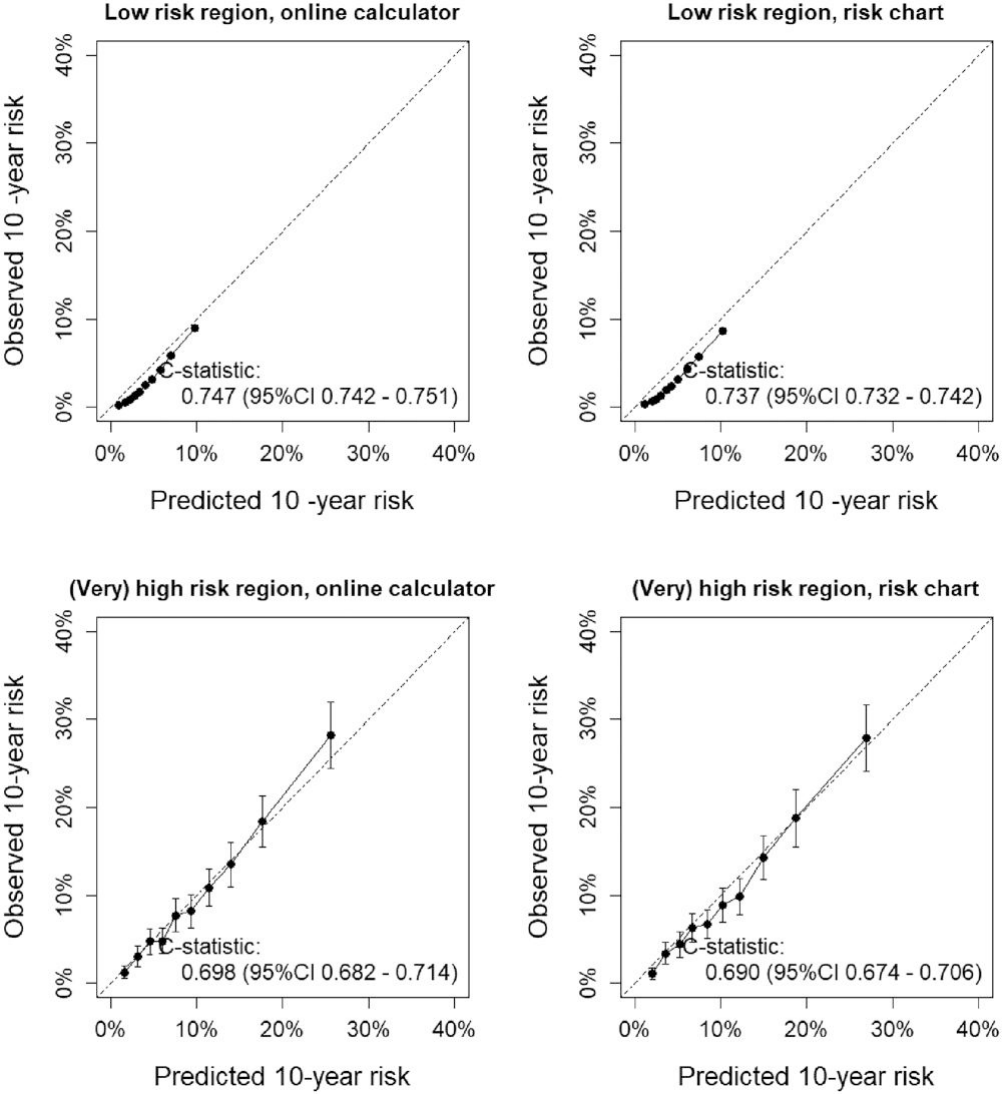
Model performance when using SCORE2 as an online calculator or with a risk chart in low and high/very high risk regions. Calibration of both methods of applying SCORE2, corrected for competing risks. The error bars in the low risk region fall within the point estimate.

In the low CVD risk region, the predicted risks were slightly higher for the risk charts (median 4.0%, IQR 2.0–6.0) vs. the online calculator (median 3.7%, IQR 2.3–5.8). Similarly, in the high/very high CVD risk region, the median risk based on the risk charts was 9.0% (IQR 5.0–15.0), vs. 8.4% (IQR 4.5–13.9) for the online calculator (all *P* < 0.001). Differences for single individuals could be substantial and increased with increasing predicted risk (see Supplementary material online, *Figure S1*). Both the risk charts and online calculator led to predictions that were well in line with observed cumulative incidence (*Figure 1*). Both the rounding of age in the risk chars, as well as the combining of HDL and total cholesterol to non-HDL-c contributed to the higher risks with the risk charts (see Supplementary material online, *Table S1*).

Discrimination was higher when the model was applied as online calculator in both low- and high/very high CVD risk regions. In the low risk region, the C-index was 0.747 (95% CI 0.742–0.751) vs. 0.737 (95% CI 0.732–0.742), with a difference in C-index of +0.010 (95% CI 0.008–0.012). In the high/very high-risk region, the C-index was 0.698 (95% CI 0.682–0.714) vs. 0.690 (95% CI 0.674–0.706), with a difference in C-index of +0.008 (95% CI 0.005–0.010). Differences in discrimination were consistent by sex (*Table 2*). Reclassification results are shown in Supplementary material online, *Table S2*.

**Table 2:** Difference in discrimination between applying SCORE2 as a risk chart or online calculator

	Calculator C-index (95% CI)	Chart C-index (95% CI)	Difference in C-index (95% CI)
Low risk region			
Overall	0.747 (0.742–0.751)	0.737 (0.732–0.742)	0.010 (0.008–0.012)
Men	0.711 (0.706–0.717)	0.702 (0.696–0.708)	0.009 (0.007–0.012)
Women	0.761 (0.754–0.768)	0.748 (0.741–0.756)	0.013 (0.010–0.016)
High/very high risk region			
Overall	0.698 (0.682–0.714)	0.690 (0.674–0.706)	0.008 (0.005–0.010)
Men	0.664 (0.641–0.686)	0.656 (0.634–0.679)	0.007 (0.004–0.010)
Women	0.713 (0.687–0.738)	0.705 (0.679–0.730)	0.008 (0.004–0.012)

Discrimination was based on Harrell’s C-statistic, corrected for competing risks.

### Clinical impact

**Figure 2:**
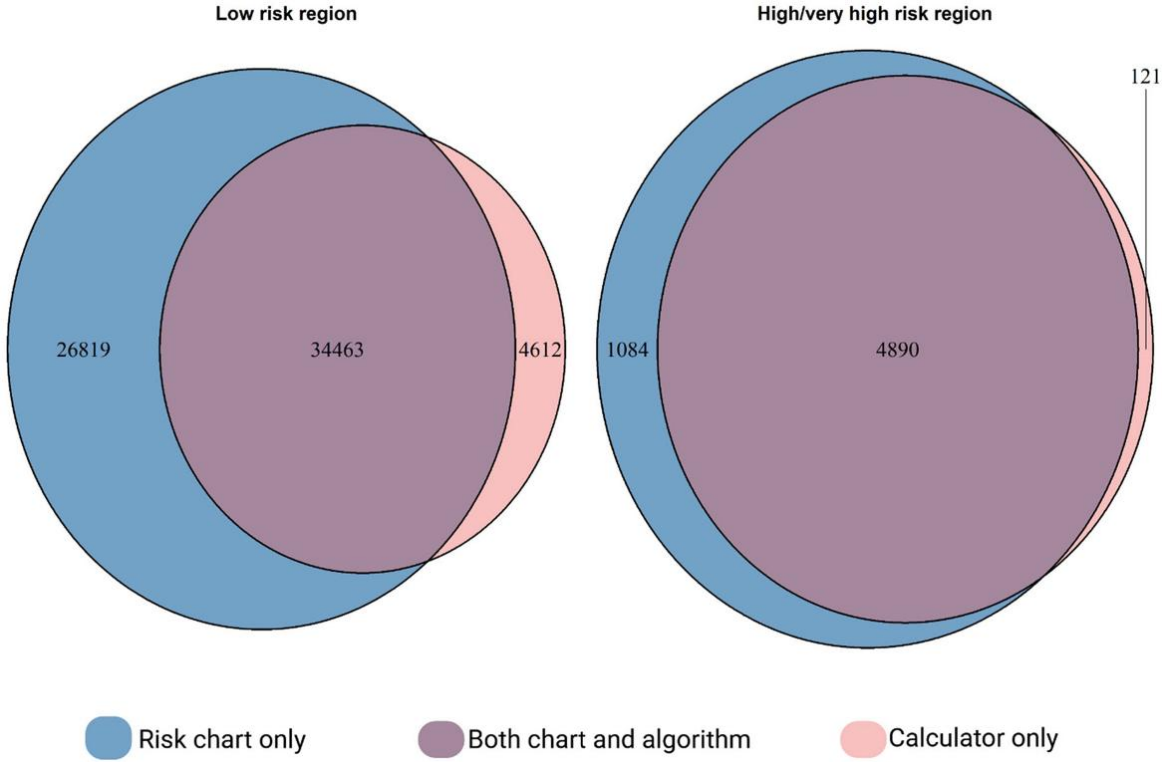
Treatment eligibility from either of the methods of applying SCORE2 for populations from low and high/very high risk region. Overlap in treatment eligibility when applying SCORE2 as risk chart vs. an online calculator, based on the treatment thresholds in the 2021 European Society of Cardiology Cardiovascular Disease prevention guidelines.^2^

In the low-risk region, 61 282 individuals (6.3%) were deemed eligible for treatment according to the risk charts, compared with 39 075 (4.0%) using to the online calculator. In the high/very high risk region, 5974 individuals (51%) were deemed eligible with the risk charts and 5011 (43%) with the online calculator. The risk charts and the online calculator largely identified the same individuals as being treatment eligible (*Figure 2*). Treatment patterns were similar for both sexes (see Supplementary material online, *Table S3*). The cumulative incidence of CVD events was highest among individuals deemed eligible for treatment by both methods, followed by individuals identified as eligible only by the online calculator (see Supplementary material online, *Figure S2*).

In the low-risk region, among the 61 282 individuals eligible for treatment according to the risk charts, there were 4962 CVD events recorded over 10 years, such that 2841 CVD events could hypothetically be prevented if preventive intervention reducing risk by 50% were offered (46 events prevented per 1000 individuals treated). Using the online calculator, the corresponding estimate was 2061 events prevented among 39 075 treated individuals (53 events prevented per 1000 individuals treated). In the high/very high risk region, treatment based on the risk charts could hypothetically prevent 74 events per 1000 individuals treated, while treatment based on the online calculator could prevent 80 per 1000 treated (*Table 3*). The net benefit of treatment with the calculator was 1.69 per 1000 people treated in the low risk region and 0.07 per 1000 people treated in the very high risk region, which was stable across different weighting factors (see Supplementary material online, *Figure S3*).

**Table 3:** Expected cardiovascular disease event reduction from both methods of applying SCORE2

	Online calculator	Risk chart
Low risk region (*n* = 977 614)		
Eligible for treatment	39 075 (4.0%)	61 282 (6.3%)
Events prevented	2061	2842
Events prevented per 1000 treated	53	46
High/very high risk region (*n* = 11 739)		
Eligible for treatment	5011 (42.7%)	5974 (50.9%)
Events prevented	403	441
Events prevented per 1000 treated	80	74

Absolute event reduction and number needed to treat were calculated based on a 10-year horizon, based on the observed CVD incidence in the cohorts among treatment-eligible individuals.

CVD, cardiovascular disease.

## Discussion

In contemporary European cohorts, the estimated 10-year CVD risks using SCORE2 were systematically higher with a CVD risk chart than those estimated with an online calculator. Higher estimated CVD risks led to a higher proportion of people eligible for preventive treatment using the age-specific risk thresholds outlined in the 2021 ESC Prevention Guidelines. Furthermore, the model’s discriminative performance was increased when using the online calculator compared with risk charts.

Multiple factors contribute to the higher predicted risks when using the SCORE2 risk charts compared with the online calculator. One major reason is the way age is handled in risk calculations. In risk charts, predictions are based on the midpoint of each 5-year age group, meaning individuals aged 60–64 years are all assigned a reference age of 62.5 years. In contrast, the online calculator is typically filled in based on an individual’s ‘age’, which in practice means age rounded down to the nearest whole number, so a person aged 64 years and 9 months will be considered 64 years old. On a population level, this translates to people being considered 6 months older in the risk charts. Another contributing factor is how cholesterol values are incorporated into the models. The SCORE2 algorithm has a separate coefficient for HDL-c and total cholesterol, which are both required for filling in the online calculator. The risk charts on the other hand require HDL-c and total cholesterol to be combined into a single non-HDL-c value. This results in individuals with different combinations of HDL-c and total cholesterol ending up with the same predicted risk (with otherwise equal risk factor levels). For example, someone with 6.0 mmol/L total cholesterol and 1.5 mmol/L HDL-c would have the same predicted risk as someone with 5.5 mmol/L total cholesterol and 1.0 mmol/L HDL-c, since both result in a non-HDL-c value of 4.5 mmol/L. However, the predictive effect of a 0.5 mmol/L increase in non-HDL-c is not the same as a 0.5 mmol/L increase in total cholesterol. This leads to individuals with relatively high HDL-c having slightly higher predicted risks when using the risk charts, while those with lower HDL-c values may have lower predictions. At the population level, this results in slightly higher overall risk estimates when using the chart compared with the online calculator.

In the 2021 ESC CVD prevention guidelines, the application of either method of SCORE2 to predict 10-year risk is recommended interchangeably: ‘The SCORE2 algorithm can be accessed in the ESC CVD Risk app (freely available from app stores) and in risk charts for the four clusters of countries.’^2^ No specific advantages or disadvantages are mentioned of either of the methods, whereas the current study reveals some differences in model performance, expected treatment eligibility rates, and overall better targeting of treatment to those at increased risk with the online calculator. For comparison, the difference in discrimination between applying SCORE2 as online calculator vs. the risk charts is comparable with the gain in discrimination that could be gained from measuring biomarkers like C-reactive protein, NT-ProBNP, or troponin-T on top of SCORE2.^14-16^ The differences in treatment eligibility are especially striking for the low risk region, where 50% more individuals would be considered eligible for treatment when using the risk charts vs. the online calculator.

To our knowledge, no direct comparison of the different methods of applying SCORE2, nor its predecessor, the SCORE model, has been conducted in terms of performance or clinical impact. However, our findings align with previous research demonstrating that grouping predicted risks into broad categories reduces predictive performance and may lead to a loss of clinically relevant information.^17^ Individuals within the same category may have significantly different risk levels, potentially affecting treatment decisions and overall risk stratification. This highlights a limitation of simplified risk classification approaches. Therefore, our study adds valuable new evidence on the practical implications of how SCORE2 is applied in routine care, emphasizing the need for careful consideration of risk estimation methods to ensure optimal clinical decision-making. Importantly, we demonstrate the magnitude of these clinical differences when comparing SCORE2 charts and the online calculator in large, real-world populations representative of different European risk regions.

Apart from the differences in model performance, practical considerations may influence the choice between the two methods of applying the SCORE2 model. The risk charts are simple in design and easy to use, making them a fast and convenient option in clinical practice. Additionally, they do not require a computer, as a paper copy can be used directly, which is especially relevant in low-resource settings such as some countries were SCORE2-ASIA is recommended.^18^ However, since in practice most consultations take place next to a computer, entering risk factors into an online calculator is a viable and often practical alternative. If electronic health records allow for automatic loading of patient data into the calculator, the calculator could be even faster than using a two-dimensional chart, as the clinician would only need to verify the input data. Real-time connection of online calculators to electronic health records could pave the road for widespread use of more complex models including those built with advanced machine learning techniques. However, challenges in implementation and transparency have so far limited their widespread adoption.^19^

Another advantage of the online calculator is the ability to estimate treatment effects, such as absolute risk reduction, which may even more effectively identify individuals who benefit most from preventive treatment. Apart from the medical device www.U-Prevent.com for CVD risk prediction, this is also possible for other diseases, such as for breast cancer patients (PREDICT tool, https://breast.predict.cam).^20^ Additionally, online calculators can accommodate a greater number of risk predictors. Even within the SCORE2 model, which includes only six risk factors, total cholesterol and HDL-c had to be combined into non-HDL-c cholesterol to fit all risk categories onto a single page.^3^ In contrast, digital tools allow for more complex models, such the SMART2 and EUROASPIRE risk calculators which also include up to 15 predictors relevant to individuals with established ASCVD.^21,22^ Similarly, machine learning-based alternatives generally require a digital interface as these are often based on a large number of predictors.

In sum, both methods of applying SCORE2 may have their own advantages in the shared decision-making process, and their usefulness may also depend on how they are implemented in practice. Since numerical risk predictions can be difficult for patients to interpret, the colour-coded risk categories in the risk charts, indicating low, moderate, or high risk, can provide a clear visual aid. Additionally, digital tools such as the ESC CVD Risk Prediction app and the U-Prevent medical device use graphical elements, such as a risk metre or bar, to indicate the level of risk. A non-coloured risk bar, such as the one used in the U-Prevent medical device, can support shared decision-making by allowing the patient and physician to collaboratively determine an appropriate risk threshold. This flexibility may enhance patient engagement and personalized treatment decisions and better respect patient autonomy.^23,24^ Future research on this aspect may further guide healthcare professionals on the choice the different methods of application.

A key strength of this study is the evaluation within robust, population-based studies. By utilizing diverse data sources from various European regions—ranging from low- to very high-risk areas—we demonstrated a consistent pattern across Europe, regardless of which risk assessment method is used. There are also limitations to consider. First, in our analyses, treatment eligibility was modelled using fixed cut-offs. In clinical reality, treatment eligibility is influenced by multiple factors, including expected side effects, patient preferences, co-morbidity, and frailty. This is particularly relevant for individuals with predicted risks close to treatment thresholds, where decisions may deviate from strict guidelines. However, as these deviations may occur in either direction, we expect that this does not importantly affect the results from the current study. Second, our analyses assumed that risk charts were applied exactly as intended. In practice, healthcare professionals may adjust their assessments for individuals with ‘outlying’ risk factors. For instance, if a patient has a systolic blood pressure or cholesterol level at the higher end of the range specified in the chart, a clinician might interpret the risk as slightly greater and be more inclined to initiate treatment. These subtle adjustments were not captured in our model, but as these might work either way, we expect that this does not have a major influence on our results. Third, the data used for the current study had follow-up ranging to 2018. Whereas this could affect the absolute risks observed in the current study due to variations in CVD incidence, it is unlikely that this affects the difference between the SCORE2 appliances. In addition, because the current analyses were solely based on European data, caution is warranted when extrapolating these findings to other models or regions, such as SCORE2-ASIA.^18^ Nevertheless, as recalibration generally has only limited impact on model discrimination, similar differences may be observed in those settings as well.

In conclusion, SCORE2 risk assessment with risk charts yields too high predicted risks, resulting in a substantially higher number of individuals eligible for treatment. Due to the loss of information with rounding off of risk factors that is required for the risk charts, the online calculator has better discriminative performance. These differences highlight the potential trade-offs between ease of use and precision when applying SCORE2 in clinical practice.

## Supplementary Material

qcaf122_Supplementary_Data

## Data Availability

The data underlying this article were provided by representatives of all included cohorts. Data from each cohort may be shared on request to the respective representatives, depending on cohort-specific policies. R-scripts used for the current analyses will be shared on reasonable request.
