# Supplementary material for: Using SCORE2 with a risk chart or online calculator: impact on model performance, treatment eligibility, and cardiovascular disease prevention
Source: Eur Heart J Qual Care Clin Outcomes. 2025 Oct 10;12(2):213–20. doi: 10.1093/ehjqcco/qcaf122 (PMC13016803; doi:10.1093/ehjqcco/qcaf122)
Supplement: qcaf122_Supplementary_Data [file qcaf122_supplementary_data.docx]

**Online supplement**

**Supplementary Figure 1: Comparison of individual predictions with SCORE2 using either the chart or the calculator**


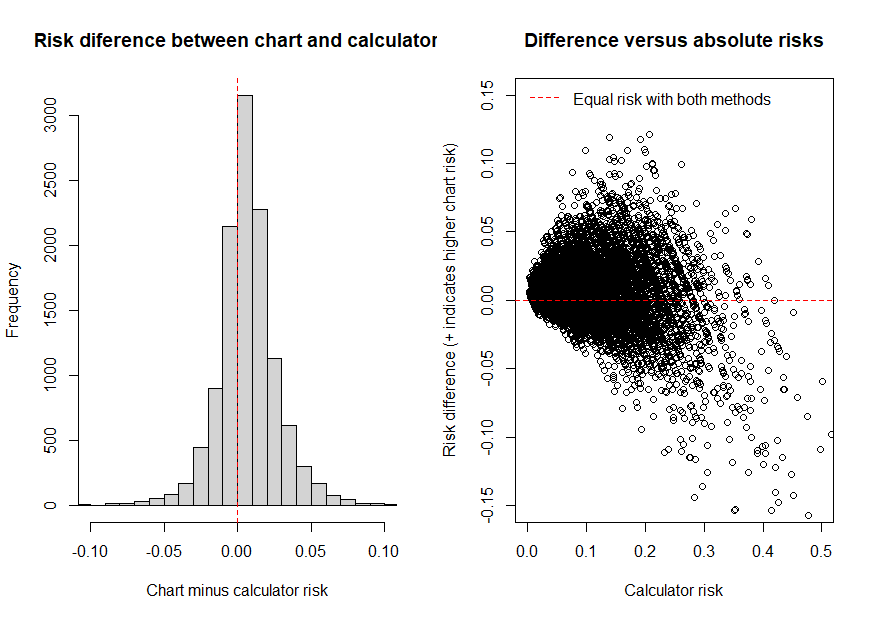


**Supplementary Figure 2: Observed CVD incidence rate among individuals identified for preventive treatment based on either or both of the methods of applying SCORE2**


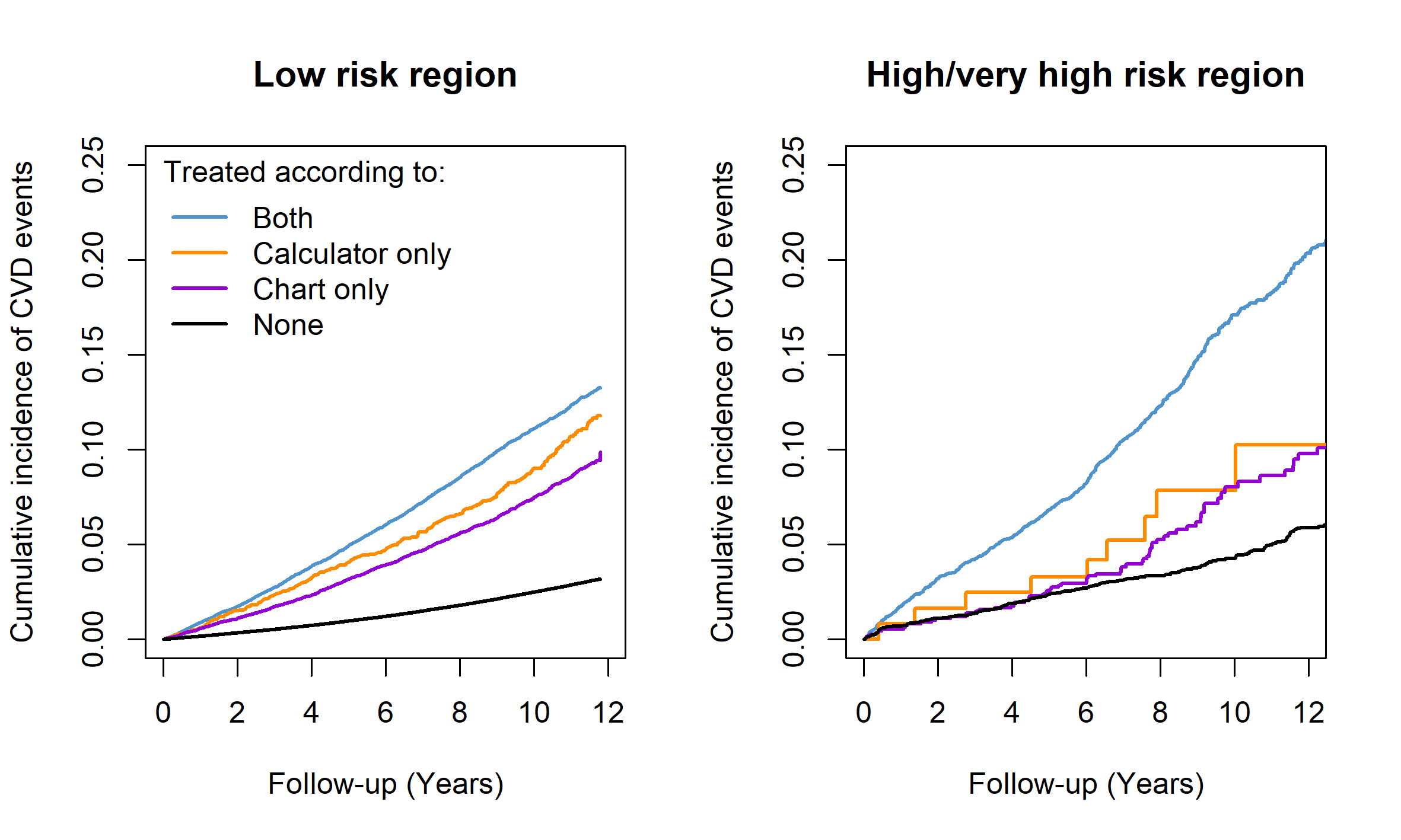


**Supplementary Figure 3: Net benefit of using an online calculator versus a risk chart, sensitivity analysis using different weighting factors**


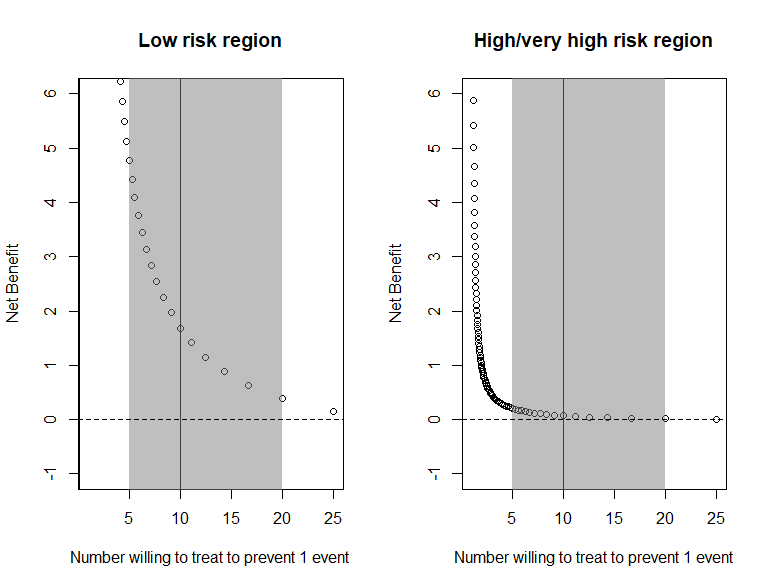


The net benefit per 1000 individuals screened is shown across different weighting factors that reflect the trade-off between treating an individual who will develop CVD (true positive) versus unnecessarily treating someone who will not (false positive). A weighting of 1:10 corresponds to the guideline-recommended treatment threshold of 10% for individuals aged 50–69 years in the ESC CVD prevention guidelines. The shaded area illustrates the sensitivity of the results when varying this trade-off, ranging from half to double the recommended weighting.

**Supplementary Table 1: Modelling steps contributing to differences in predicted risk**

| **SCORE2 method** | **Median 10-year risk, % (IQR)** |  |
| --- | --- | --- |
| Risk charts | 8.4% (4.5-13.9) |  |
| Online calculator | 9.0% (5.0-15.0) |  |
| *Online calculator with rounding* | 8.4% (4.5-13.7) |  |
| *Online calculator with age + 0.5 year* | 8.7% (4.7-14.3) |  |
| *Online calculator using non-HDL* | 8.9% (5.0-14.5) |  |

Comparison of the different components that make up the difference between the unrounded algorithm and the charts, example in the high/very high risk region:

A) Online calculator with rounding: Applies rounding to all risk factors as done in the risk charts, while keeping the mean age constant and using separate values for HDL and total cholesterol.

B) Online calculator with age +0.5 years: Simulates the effect of age rounding on the risk charts by uniformly increasing participants’ age by 0.5 years (see Discussion section for rationale).

C) Online calculator using non-HDL: Substitutes total and HDL cholesterol with non-HDL cholesterol to mirror the risk chart calculation method, without rounding other risk factors.

**Supplementary Table 2: Reclassification of applying SCORE2 as online calculator versus risk chart**

| **Population** | **Event** | **Non-event** | **Number reclassified** |
| --- | --- | --- | --- |
| Low risk region | -0.032 (-0.037 to -0.027) | 0.031 (0.031 to 0.032) | 134,581 (14%) |
| High/very high risk region | -0.055 (-0.078 to -0.035) | 0.098 (0.091 to 0.106) | 1884 (16%) |

The net reclassification index (NRI) quantifies the extent to which one model more appropriately reassigns individuals to different risk categories compared with another model. The event NRI reflects whether individuals who experienced a cardiovascular event were more often reclassified upward into a higher risk category (positive values) or downward into a lower risk category (negative values) when using the online calculator compared with the risk chart. The non-event NRI reflects whether individuals who did not experience an event were more often reclassified downward (positive values) or upward (negative values). For the current analysis, NRI was calculated using cut-offs of 5% and 10% 10-year risk, in line with current treatment thresholds. Positive numbers indicate better performance of the online calculator.

**Supplementary Table 3: Treatment eligibility when applying SCORE2 as online calculator versus risk chart according to sex**

|  | **Online calculator** | **Risk chart** |
| --- | --- | --- |
| *Low risk region* |  |  |
| Men | 33880 (7%) | 51638 (11%) |
| Women | 5195 (1%) | 9644 (2%) |
|  |  |  |
| *High/very high risk region* |  |  |
| Men | 2989 (57%) | 3492 (67%) |
| Women | 2022 (31%) | 2482 (38%) |

Individuals identified as eligible for treatment (i.e. with a predicted risk higher than the respective treatment target) according to the ESC CVD Prevention guidelines: ≥7.5% 10-year risk for individuals aged 40-49 years, ≥10% 10-year risk for individuals aged 50 to 69 years. Numbers represent the absolute count and percentage of individuals within each sex and risk region classified as treatment-eligible.
